# Agreement between bovine respiratory disease scoring systems for pre-weaned dairy calves

**DOI:** 10.1017/S1466252314000164

**Published:** 2014-11-26

**Authors:** Sharif S. Aly, William J. Love, Deniece R. Williams, Terry W. Lehenbauer, Alison Van Eenennaam, Christiana Drake, Philip H. Kass, Thomas B. Farver

**Affiliations:** 1 Veterinary Medicine Teaching and Research Center, School of Veterinary Medicine, University of California, Davis, Tulare, California, USA; 2 Department of Population Health and Reproduction, School of Veterinary Medicine, University of California, Davis, California, USA; 3 Department of Animal Science, University of California, Davis, California, USA; 4 Department of Statistics, University of California, Davis, California, USA

**Keywords:** bovine respiratory disease, dairy calves, clinical signs, CA scoring system, WI scoring system

## Abstract

Clinical scoring systems have been proposed for respiratory disease diagnosis in calves, including the Wisconsin (WI) system (McGuirk in 2008) which uses five clinical signs, each partitioned into four levels of severity. Recently, we developed the California (CA) bovine respiratory disease (BRD) scoring system requiring less calf handling and consisting of six clinical signs, each classified as normal or abnormal. The objective of this study was to estimate the on-farm agreement between the WI and the CA scoring systems. A total of 100 calves were enrolled on a CA dairy and assessed for BRD case status using the two scoring systems simultaneously. The Kappa coefficient of agreement between these two systems was estimated to be 0.85, which indicated excellent agreement beyond chance. The simpler design and reduced calf handling required by the CA BRD scoring system may make it advantageous for on-farm use.

## Introduction

Bovine respiratory disease (BRD) is an important disease of calves that leads to reduced weight gain and productivity (Waltner-Toews *et al*., 1986; Warnick *et al*., 1997; Donovan *et al.*, 1998). Field diagnosis remains a challenge for control and treatment of BRD. Several clinical scoring systems have been proposed, including the Wisconsin (WI) system (McGuirk, 2008) that uses five clinical signs, each partitioned into four levels of severity. Recently, we developed a BRD scoring system for pre-weaned dairy calves based on a matched case–control study of 2030 Holstein calves on a large calf ranch in the San Joaquin Valley, California (CA) (BRD3 scoring system; Love *et al*., 2014). The CA BRD scoring system is based on the presence or absence of six clinical signs, each assigned a model-based score (Appendix 1). The objective of this study was to estimate the on-farm agreement between the WI and the CA BRD scoring systems in pre-weaned dairy calves.

## Materials and methods

Agreement beyond chance between the two systems was estimated using Kappa (κ), as described by Cohen (1960). Given a 1:3 allocation between BRD cases and non-cases, a sample size of 100 calves was required to detect a true κ value of 0.75 in a two-sided test of the null hypothesis that κ is equal to 0 with 99% power and 5% level of significance (Flack *et al*., 1988). The κ estimate was interpreted as poor if <0.40; fair to good if 0.41–0.75; or excellent agreement beyond chance if >0.75 (Fleiss *et al.*, 2003).

One hundred pre-weaned calves from a 3200 dairy cow herd were evaluated by the same investigator for clinical signs required by the WI and CA scoring systems, including nasal discharge, ocular discharge, cough (spontaneous and induced), respiration quality, temperature, and ear droop or head tilt. Study personnel identified ill calves with signs of depression, anorexia, or loss of appetite which were then examined by thoracic auscultation and ultrasound. Calves with abnormal ultrasound or auscultation were identified as cases and scored using both the WI and CA scoring systems. In addition, a random sample of approximately five to ten healthy calves were enrolled and scored using both systems on study days.

The study calves were housed in individual wooden hutches with elevated slatted floors and given free choice water and starter grain until weaning at approximately 75 days of age. Calves were fed 2 liters of pasteurized waste milk supplemented with milk replacer containing 20% protein and 20% fat twice a day. In the first month of life, calves were treated with an intra-nasal, modified live vaccine for the prevention of infectious bovine rhinotracheitis virus, bovine respiratory syncytial virus, and parainfluenza type 3 virus; and a parenteral, killed vaccine for the above viruses and bovine viral diarrhea virus types 1 and 2. Data analysis was performed using Stata 13.1 (College Station, Texas, USA).

## Results

Of the 100 calves enrolled, 63 were Holsteins and 37 were Jerseys. Sixty-eight calves were heifers. Enrolled calves ranged from 21 to 110 days of age with a mean age of 62 days. The CA scoring system identified 29 calves positive for BRD, and the WI system identified 27 calves positive for BRD. Twenty-five calves were identified as cases by both scoring systems. Cohen's κ for the two scoring systems was estimated to be 0.85 (SD = 0.099) indicating that the WI and CA BRD scoring systems had excellent agreement beyond chance.

## Significance

Agreement beyond chance between the WI and the CA scoring systems based on 100 pre-weaned calves on a CA dairy was excellent. Calf raisers assessing BRD in pre-weaned calves on dairies with similar management conditions and risk for BRD can use the CA scoring system and expect excellent agreement with the WI scoring system. However, differences exist between the two systems. The WI scoring system assesses five clinical signs including ocular discharge, nasal discharge, induced or spontaneous cough, rectal temperature, and head tilt or ear position. In addition to these five clinical signs, the CA system assesses quality of respiration but involves less calf handling as it does not require additional handling of calves for inducing a cough, which may be a biosecurity concern due to the potential transfer of infectious material between calves. Another difference is that the WI scoring system relies on four severity levels for each clinical sign with scores uniformly distributed between 0 and 3. In contrast, the CA system relies on a simpler design, the presence or absence of clinical signs, and utilizes model-based scores. As a result, using the CA scoring system, only calves with either nasal discharge or any two clinical signs of spontaneous cough, ocular discharge, or abnormal respiration would require calf handling to measure rectal temperature and confirm BRD status. The simpler design and reduced calf handling required by the CA scoring system may make it the preferred scoring system for on-farm use.

Limitations of the current study include the use of a single herd and the cross-sectional study design. Hence, the agreement between the WI and CA BRD scoring system may change in herds with different calf management conditions and BRD risk. Future research should describe the diagnostic accuracy (sensitivity and specificity) of such scoring systems in order to better inform users about the strengths and weaknesses of using scoring systems for on-farm diagnosis of BRD in calves.

## Appendix

**Appendix 1.** The California Bovine Respiratory Disease (BRD) scoring system for pre-weaned dairy calves^1^ Add scores for all six signs, if total score is ⩾5, calf may be a BRD case.

Clinical sign	Score if normal		Score if abnormal (any severity)^2^			
Eye discharge	0		2	Or		Or
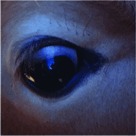
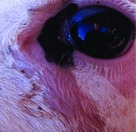
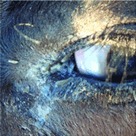
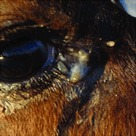
Nasal discharge	0		4	Or		Or
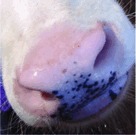
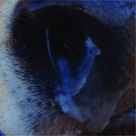
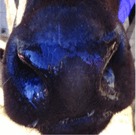
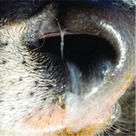
Ear droop/Head tilt	0		5	Or		Or
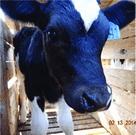
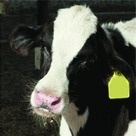
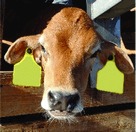
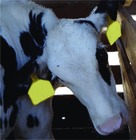
Cough	0	No cough	2	Spontaneous cough		
Breathing	0	Normal	2	Rapid or difficult breathing		
Temperature	0	<39.2°C	2	⩾39.2°C (102.5°F)		

1 BRD3 scoring system in ‘Development of a novel clinical scoring system for on-farm diagnosis of bovine respiratory disease in pre-weaned dairy calves.’ Love *et al.* (2014). https://peerj.com/articles/238.pdf

2 Any abnormality including, but not limited to, the examples shown in the above pictures.
